# An examination of imaging findings in patients with clinically diagnosed gluteal tendinopathy: a secondary analysis of a randomised clinical trial

**DOI:** 10.1007/s00402-025-05964-z

**Published:** 2025-06-19

**Authors:** Alison Grimaldi, Anthony Nasser, Rebecca Mellor, Bill Vicenzino

**Affiliations:** 1 University of Queensland, Brisbane, Australia; 2 University of Technology Sydney, Sydney, Australia; 3 Gallipoli Medical Research Foundation, Greenslopes, Australia

**Keywords:** Gluteal tendinopathy, Gluteal tendon tears, Hip, Buttocks, GTPS, Trochanteric bursitis

## Abstract

**Objectives:**

To report tendon pathology present on imaging and relationships to pain, function and disability in participants with gluteal tendinopathy (GT) who participated in a multi-centre randomised clinical trial.

**Materials and methods:**

Imaging findings were examined in 204 participants with GT. Magnetic Resonance Images (MRI) and x-ray were evaluated against pre-determined criteria by experienced radiologists, blind to clinical findings, who reported on location of tendon pathology, presence and severity of tendon tears and location and presence of calcification. Severity of changes seen on MRI were scored and analyses for relationships to pain, function and disability were performed.

**Results:**

Information on location and severity of tendon pathology was available for 202 (99%) of the 204 LEAP trial participants. Tendon pathology was commonly present in both the gluteus medius (GMed) and gluteus minimus (GMin) tendons (130/202; 64%), with tears in one or both tendons in 85 (42%) participants. Of 99 tears across both tendons, 77 (78%) were partial-thickness and 22 (22%) were full-thickness in nature. The anterior aspect of the GMed tendon and the posterior aspect of the GMin tendon were most afflicted. Calcifications at the lateral ilium or greater trochanter were evident on x-ray in 73 (36%) participants. Pain, function and disability were not associated with the severity of changes on MRI.

**Conclusion:**

In a clinical trial of people with GT, a substantial number of participants had multiple tendons affected, as well as concomitant tendon tears and calcification. MRI detected changes were not associated with levels of pain, function or disability.

**Supplementary Information:**

The online version contains supplementary material available at 10.1007/s00402-025-05964-z.

## Introduction

Gluteal tendinopathy (GT) is prevalent in women over 40 years, and is the most common lower limb tendinopathy, with an estimated incidence of 35 per 1000 person-years [1]. The key feature of the condition is lateral hip pain, which results in sleep disruption and difficulty in the performance of weight-bearing activities, such as walking and stair-climbing [2].

Imaging is often performed in those with trochanteric pain to assist diagnosis and treatment selection [3]. It has been recommended that treatment for GT should be staged depending on severity of imaging findings, with platelet-rich-plasma suggested for tendinopathy or partial-tears, and surgical intervention suggested for full-thickness gluteal tendon tears and partial-thickness tears not responding to platelet-rich-plasma injections [3]. The high prevalence of pathological changes in the gluteal tendons of people without trochanteric pain [4] challenges such imaging-based treatment recommendations of invasive treatments. This potentially impacts clinical practice, where an imaging report of a tendon tear or calcification may lead the patient or clinician to assume that some form of invasive procedure will be necessary to repair the tendon. Clinicians would likely benefit from reports of imaging findings in participants who have not undergone invasive treatments such as platelet-rich-plasma injections or surgery for their GT [5].

Baseline imaging data from a randomised clinical trial (RCT) of non-surgical treatments may provide clinicians with useful information on how to interpret imaging findings in patients with GT and assist with evidence-based management planning [5]. The trial reported the efficacy of education-and-exercise compared to corticosteroid injection (CSI) and wait-and-see for management of symptomatic GT, diagnosed clinically and independently confirmed with Magnetic Resonance Imaging (MRI) [5]. The education-and-exercise approach returned highest rates of patient-reported global improvement at 8 and 52 weeks (near-80% success), and greatest pain reduction at 8 weeks [5]. Participants who received CSI reported greater pain relief than the wait-and-see group at 8 and 52 weeks, and better global improvement at 8 weeks, but not 52 weeks [5].

This prospective observational secondary analysis of RCT data examined the type and extent of imaging-detected changes in the tendons of gluteus medius (GMed) and gluteus minimus (GMin) muscles in participants with GT included in a multi-centre RCT [5]. It also examined if the severity of trochanteric soft-tissue pathology rated on MRI is associated with patient features of pain, function and disability.

## Methods

### Design

This prospective observational secondary analysis of RCT data examined data from a three-arm RCT involving 204 participants randomised to receive education-and-exercise, ultrasound-guided CSI or a wait-and-see approach. The RCT was prospectively registered (ACTRN12612001126808), approved by the institutional ethics committees (#2012000930; ID 1238598) and its primary analyses reported previously [5]. The primary LEAP trial was funded by a National Health and Medical Research Council (NHMRC) programme grant (#631717).

### Participants

Participant eligibility criteria of the RCT has been described in detail previously [6], with all participants testing positive on clinical tests for GT and demonstrating pathological changes on MRI within the GMed and/or GMin tendons of insertion at the greater trochanter. At a minimum, intratendinous increase in signal intensity on T2-weighted images was required for inclusion.

Two hundred and four participants (167 females) were included and randomised to either education-and-exercise (*n* = 69), a single ultrasound-guided CSI (*n* = 66) or a wait-and-see approach (*n* = 69). Participants’ mean age was 54.8 (± 8.8) years. Further demographic details have been previously reported [5].

### Interventions

The education-and-exercise group consisted of 14 physiotherapist-led sessions over the course of eight weeks. Education focused on self-management strategies including understanding aggravating and easing factors and the importance of graded progression of tendon loading in recovery. Exercise focused on progressive loading of the hip abductor musculotendinous unit and improving dynamic control of hip adduction during functional activities. In addition to a daily home exercise routine, the exercise program was supervised by a physiotherapist in a clinic twice per week. The CSI group received a single dose of Celestone (1 ml) or Kenacort-A 40 (1 ml) and local anaesthetic, injected under ultrasound guidance into the trochanteric bursa by an experienced radiologist. The wait-and-see group consisted of a single session with a physiotherapist who provided general information about the condition, advice regarding activity levels and reassurance regarding the prognosis of the condition. Additional information regarding interventions can be found elsewhere [6].

### Primary imaging outcomes

The outcomes of interest in this current report were location of tendon pathology, prevalence, severity and location of tendon tears, and frequency of calcifications at the greater trochanter or lateral iliac crest.

### Imaging examination

Participants underwent radiographic and MRI examinations to determine study eligibility. All images were examined by an experienced radiologist who was blind to clinical examination findings. Radiologists had on average 8.5 (4–11) years of experience post specialty training. Radiographs (antero-posterior and lateral x-rays) were taken to grade hip osteoarthritis severity using the Kellgren-Lawrence Scale [7]. Those with a score of > 2, indicating more than minimal radiographic osteoarthritis, were excluded from the study. This data is available in supplementary Table 1. The presence of calcifications at the greater trochanter, both superolateral and inferior, as the GMed tendon blends into the superior tendon of origin of the vastus lateralis [8], and calcifications at the lateral iliac crest, at the origin of the hip abductor musculature, were also reported as they may reflect regional entheseal changes or a broader pattern of abductor overload [9].

The MRI images were acquired on a MAGNETOM Espree 1.5 T scanner (Siemens AG), and the imaging protocol included the following sequences: axial PD fat sat (TR 3130, TE 23, 23 × 3.5 mm slices, Base res 384); coronal PD fat sat (TR 3050, TE 42, 19 × 3.5 mm slices, Base res 384); coronal T1 (TR 459, TE 12, 19 × 3.5 mm slices, Base res 384); sagittal PD fat sat (TR 3560, TE 39, 23 × 3.5 mm slices, Base res 384); sagittal PD (TR 2470, TE 31, 23 × 3.5 mm slices, Base res 384), all with an 0.7 mm×0.5 mm matrix.

### Classifying and scoring pathology on images

Radiologists received pre-specified criteria for classification of GT pathology based on a published classification system [4]. Intratendinous high T2 signal was considered as tendon pathology. The classification system is summarised in Table 1. Location of tendon pathology was recorded.

**Table 1:** Criteria for diagnosis of gluteal tendon pathology on MRI [4]

Tendon pathology without tear	Intratendinous high T2 signal was considered as tendon pathology
Partial tear	A partial-thickness tear was diagnosed if the tendon was irregular, thinned or focally discontinuous on T1-weighted images, with hyperintensity in the corresponding area on T2-weighted images
Full-thickness tear	Discontinuity and/or retraction of the torn tendon was seen on T1-weighted images, with a marked increase in signal on T2-weighted images.

The classification items were then scored using a previously reported scoring system [10]. In brief, 4 points were allocated to the degree of T2 hyperintensity signal, 3 points allocated to location of tendon pathology, 6 points to size of GMin/GMed tendon tears and 0 points if there were no abnormalities reported (Table 2).

**Table 2:** MRI pathology score [10]

Item	Criteria	Score
Normal	No change on image	0
T2 hyperintensity signal around greater trochanter	Tiny– thin slit or fluid	1
	Small– localised, mild distension	2
	Medium– localised, moderate distension	3
	Large– localised, marked distension	4
Location of tendon pathology	Gluteus minimus	1
	Gluteus medius	2
	Both	3
Tendon Tear Size:		
Gluteus minimus	Partial	1
	Full	2
Gluteus medius	Partial	2
	Full	4
Total Possible Score		13

### Pain, function and disability levels

Pain was measured on a 0–10 scale, where no pain was 0 and worse pain imaginable was 10 [10]. Function was measured with a patient specific function scale– whereby participants nominated 3 functions and rated their level on a 0–10 scale, where 0 was no function and 10 was normal function [11]. Disability was captured with the Victorian Institute of Sport Assessment– gluteal tendinopathy (VISA-G), a 0-100 scale in which 0 is worse disability and 100 is no disability [12].

### Data analysis

MRI data for all eligible participants were collated in Microsoft Excel within the prespecified classifications (e.g., severity and location of tendon pathology). Pre-specified criteria for tendon pathology without tear, partial-thickness tear and full-thickness tear are described in Table 1. Data was then tallied and expressed as percentages of the total number of participants. Any missing data was reported.

The relationship between MRI pathology score and pain, function and disability was assessed by visualisation of the scatterplots and with Pearson Correlations (*p* < 0.05) using SPSS statistical program (IBM SPSS Statistics v29.0.1.0).

## Results

### MRI findings

For the 204 participants with GT included in the trial, information on location and severity of tendon pathology was available in radiology reports for 202 (99%) participants. Specific details on location and severity of tendon pathology (presence of tears) had not been reported by the radiologist for the remaining participants. Data on the location and severity of tendon pathology on MRI is reported in Table 3.

### Location of tendon pathology

Pathology was identified in both GMed and GMin tendons in 130 (64%) participants, only in the GMed tendon in 38 (19%), and only in the GMin tendon in 34 (17%) participants.

Of the 168 participants reported to have GMed tendinopathic changes, data on the location of GMed tendon pathology (anterior, posterior or both) was available for 166 (99%). Of these, only the anterior portion of the GMed tendon had pathology in 130 (78%), only the posterior portion of the tendon had pathology in 10 (6%) and both portions were affected in 26 (16%) participants.

Of the 164 participants reported to have GMin tendinopathic changes, data on location of GMin tendon pathology (anterior, posterior or both) was available for 160 (98%). Of these, only the anterior portion of the tendon (the more ventral 50% of the tendon) had pathology in 14 (9%), only the posterior portion of the tendon (the more dorsal 50% of the tendon) had pathology in 53 (33%), and both portions were affected in 93 (58%) participants.

### Frequency of tendon tears

Tears in one or both of the gluteal tendons were reported in 85 (41%) of the 202 participants. Tendon tears were present in 34 of 69 (49%) of the education-and-exercise group, 28 of 66 (42%) of the corticosteroid group, and 23 of 69 (33%) of the wait-and-see group participants.

**Fig. 1:**
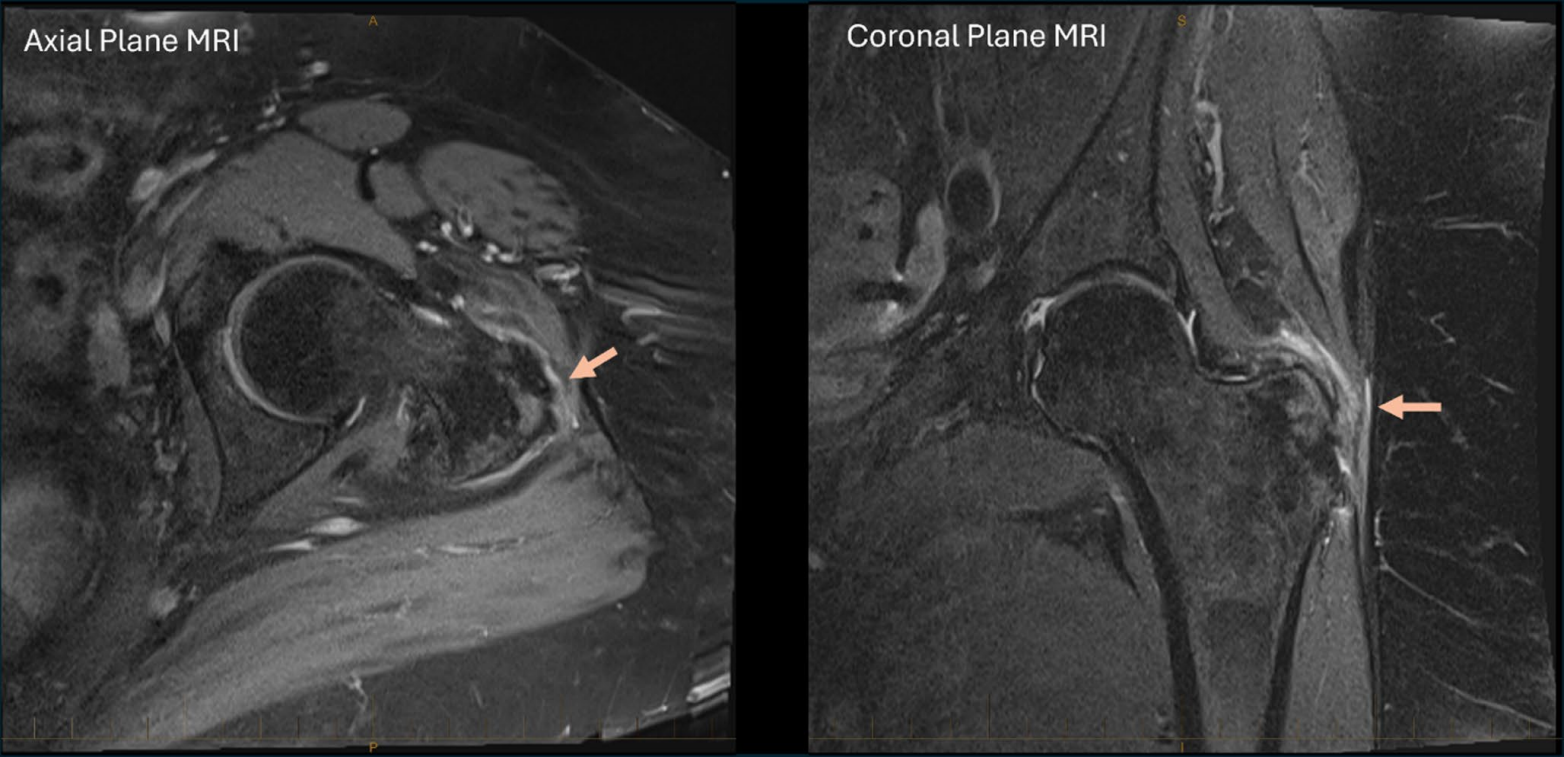
An example of tendon pathology in a participant of the LEAP trial demonstrated on axial and coronal plane T2-weighted MRI. Tears of the gluteus medius and minimus on the background of severe tendinopathy was reported

Data on the location of the gluteal tendon tears was available for 83 (98%) participants. Of those 83 participants, 40 (48%) had an isolated GMin tear, 27 (33%) had an isolated GMed tear and 16 (19%) had tears in both tendons. See Fig. 1 for an example of a LEAP trial participant with more severe tendon pathology.

### Location of tendon tears

Further information on the location of the tear within each tendon (anterior, posterior or both) was available for 39 of the 43 (91%) participants with GMed tendon tears, and 44 of the 56 (79%) participants with GMin tears. Of those with a GMed tear, a tear was present only in the anterior region of the tendon in 30 (77%), only in the posterior region of the tendon in 4 (10%), and in both aspects of the tendon in 5 (13%) participants. Of those with a GMin tear, a tear was present only in the anterior region of the tendon in 6 (14%), only in the posterior region of the tendon in 23 (52%) and in both aspects of the tendon in 15 (34%) participants.

**Table 3:** Location and severity of tendon pathology on magnetic resonance imaging

	Number of Participants *n* (%)	Total Number of Participants
**Location of Pathology**		
GMed & Min path	130 (64)	202
GMed path only	38 (19)	202
GMin path only	34 (17)	202
Unspecified/missing data*	2 (1)	204
**Location of GMed Pathology**		
Ant GMed Path only	130 (78)	166
Both Ant & Post GMed Path	26 (16)	166
Post GMed path only	10 (6)	166
Unspecified/missing data*	2 (1)	168
**Location of GMin Pathology**		
Ant & Post GMin Path	93 (58)	160
Post GMin Path only	53 (33)	160
Ant GMin Path only	14 (9)	160
Unspecified/missing data*	4 (2)	164
**Presence of Tendon Tears**		
No tendon tear	117 (58)	202
Tear in one or both tendons	85 (42)	202
**Location of Tendon Tears**		
GMin tear	40 (48)	83
GMed tear	27 (33)	83
Tear GMed and GMin	16 (19)	83
Unspecified/missing data*	2 (2)	85
**Location of GMed Tear**		
Ant GMed tear	30 (77)	39
Ant & Post GMed tear	5 (13)	39
Post GMed tear	4 (10)	39
Tear unspecified location*	4 (10)	43
**Location of GMin Tear**		
Post GMin Tear	23 (52)	44
Ant & Post GMin tear	15 (34)	44
Ant GMin Tear	6 (14)	44
Tear unspecified location*	12 (21)	56
**Severity of Tendon Tears**		
**Gluteus Medius Tears**		
P/T GMed tear	35 (81)	43
F/T GMed tear	8 (19)	43
**Gluteus Minimus Tears**		
P/T GMin tear	42 (75)	56
F/T GMin tear	14 (25)	56

### Severity of tendon tears

The severity of tendon tears was rated as either partial or full thickness. A rating of severity was available for all tendon tears identified. Tears were partial-thickness in 35 (81%) and full-thickness in 8 (19%) participants with GMed tears. For GMin tears, 42 (75%) had partial-thickness tears, while 14 (25%) had full-thickness tears (Table 2).

### MRI pathology score and its relationship to pain, function and disability

The mean (SD) for the MRI pathology score was 5.6 (2.2), pain severity 7.5 (1.3), patient specific functional scale 4.6 (1.9) and VISA-G 59.9 (12.5). There was no correlation between MRI severity score and pain, function or disability (Pearson R ranged from − 0.4 to 0.3, p-value > 0.6). Correlation matrix and statistics are in supplementary material (supplementary Table 2).

### X-ray findings

#### Frequency of calcifications

**Fig. 2:**
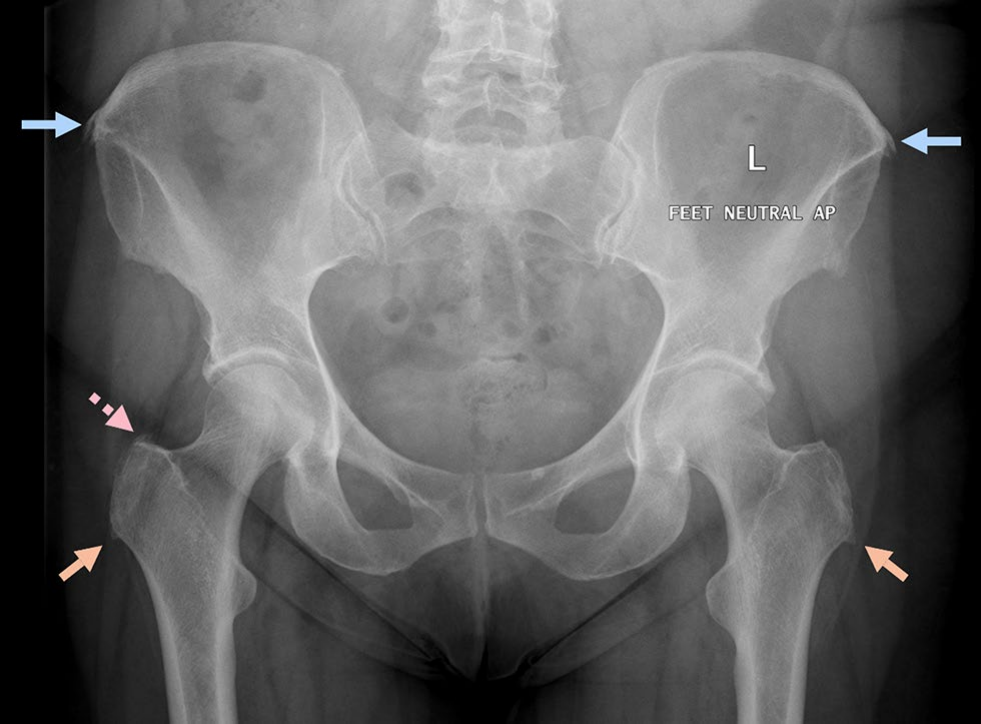
Tendon calcifications evident on radiographs. Superior/Blue arrows: Gluteus medius origin at iliac crest. Dotted/ Pink arrow: Gluteal tendons at the superolateral aspect of the greater trochanter. Inferior/Orange arrows: Calcifications extending inferiorly from the greater trochanter

Information on calcifications was available for 202 (99%) participants. Calcifications were evident on antero-posterior radiographs in 73 (36%) participants (Table 3). Calcifications were evident in three locations– at the lateral ilium in 39 (19%), the superolateral aspect of the greater trochanter in 38 (19%) and the inferior aspect of the greater trochanter in 35 (17%) participants (Table 4; Fig. 2).

**Table 4:** Frequency and location of calcifications on x-ray

Calcifications	Number of participants *n* (%)*
Frequency:	73 (36)
Location:	
Lateral ilium	39 (19)
Superolateral aspect of greater trochanter	38 (19)
Inferior aspect of greater trochanter	35 (17)

*2 (1%) not reported

## Discussion

This study details the location and severity of tendon pathology in 202 participants of the LEAP RCT, all of whom had a clinical presentation of GT confirmed by MRI. Tendon pathology was commonly present in both the GMed and GMin tendons (130; 64%), with tears occurring in one or both tendons in 85 (42%) participants and the severity of these tears being partial-thickness in 77 (78%) and full-thickness in 22 (22%) tears. Pathology and tears were more likely to occur in the anterior aspect of the GMed tendon and the posterior aspect of the GMin tendon. Calcifications at the lateral ilium or greater trochanter were also evident on x-ray in 73 (36%) participants. The severity of MRI findings was not associated with the participant’s reported pain, function or disability levels.

### Implications for selection of interventions

Many clinicians and patients interpret a finding of a gluteal tendon tear as an indicator that an orthopaedic review for surgical intervention may be required. It has been suggested that selection of interventions should be based on stage of gluteal tendon pathology, with surgical intervention recommended for full-thickness gluteal tendon tears and platelet-rich-plasma, followed by surgery as required, for partial-thickness tears [3]. In that review, the LEAP trial results were assumed to be relevant only to patients with early tendon pathology, without tears [3]. This new information on the severity of tendon pathology and its poor relationship to patient pain, function and disability, together with the secondary analysis of moderators of effects of interventions in the LEAP trial [10], suggest that the stage of pathology may not be the most important criterion for treatment selection. Tendon tears were present in 34/69, (49%) of the group who were randomised to the education-and-exercise intervention, which returned a 77–78% success rate and superiority over other interventions (CSI and wait-and-see) in terms of patient-reported global improvement at 8 and 52 weeks. The subsequent moderator analysis revealed that the severity of tendon pathology in the LEAP cohort did not significantly influence the difference in outcomes between treatments [10].

Mediation analysis has shown that treatment effects were significantly mediated by patient-rated measures of pain constancy, pain self-efficacy and function, rather than more anatomical or biological constructs [10]. This appears to align with our findings that pain and function were not correlated to the severity of pathology on MRI. Furthermore, partial-thickness tears of the GMed and GMin tendons have been reported in up to 25% of hips in people without trochanteric pain [4], and most partial-thickness gluteal tendon tears in those with symptoms do not progress over a mean of 6 years [13]. Schenk and colleagues recently reported that 90% of partial-thickness GMed or GMin tendon tears remained stable or became a scar, and only 34% of symptomatic gluteal tendon lesions progressed over this timeframe [13]. Despite their hypothesis suggesting the contrary, the authors concluded that non-operative treatment might be a valid long-term option for degenerative hip abductor lesions.

These findings suggest that surgical interventions should be reserved for those who have an unsatisfactory response to rehabilitative efforts (e.g., education and graded exercise). Clinicians and their patients should be reassured that despite partial or full-thickness gluteal tendon tears on MRI in those with trochanteric pain, positive short and long-term outcomes are possible with a non-invasive education-and-exercise approach. Patient-rated constructs may be more relevant than imaging findings in determining treatment direction and measuring effects of treatment for GT [10].

### Implications for pathomechanics and exercise approaches

Although pathological imaging findings appear to be poor predictors of both symptoms [4] and outcomes of treatment [10], absence of trochanteric soft tissue pathology makes a diagnosis of GT unlikely [4]. This suggests that tendon pathology could be considered a risk factor for the development of pain [14]. Better understanding of risk factors may assist in management strategies. The aetiology of tendon pathology is multifactorial, including factors such as age, hormonal status and systemic or metabolic factors, as well as mechanobiological factors associated with local loading of the tendon tissue [15].

**Fig. 3:**
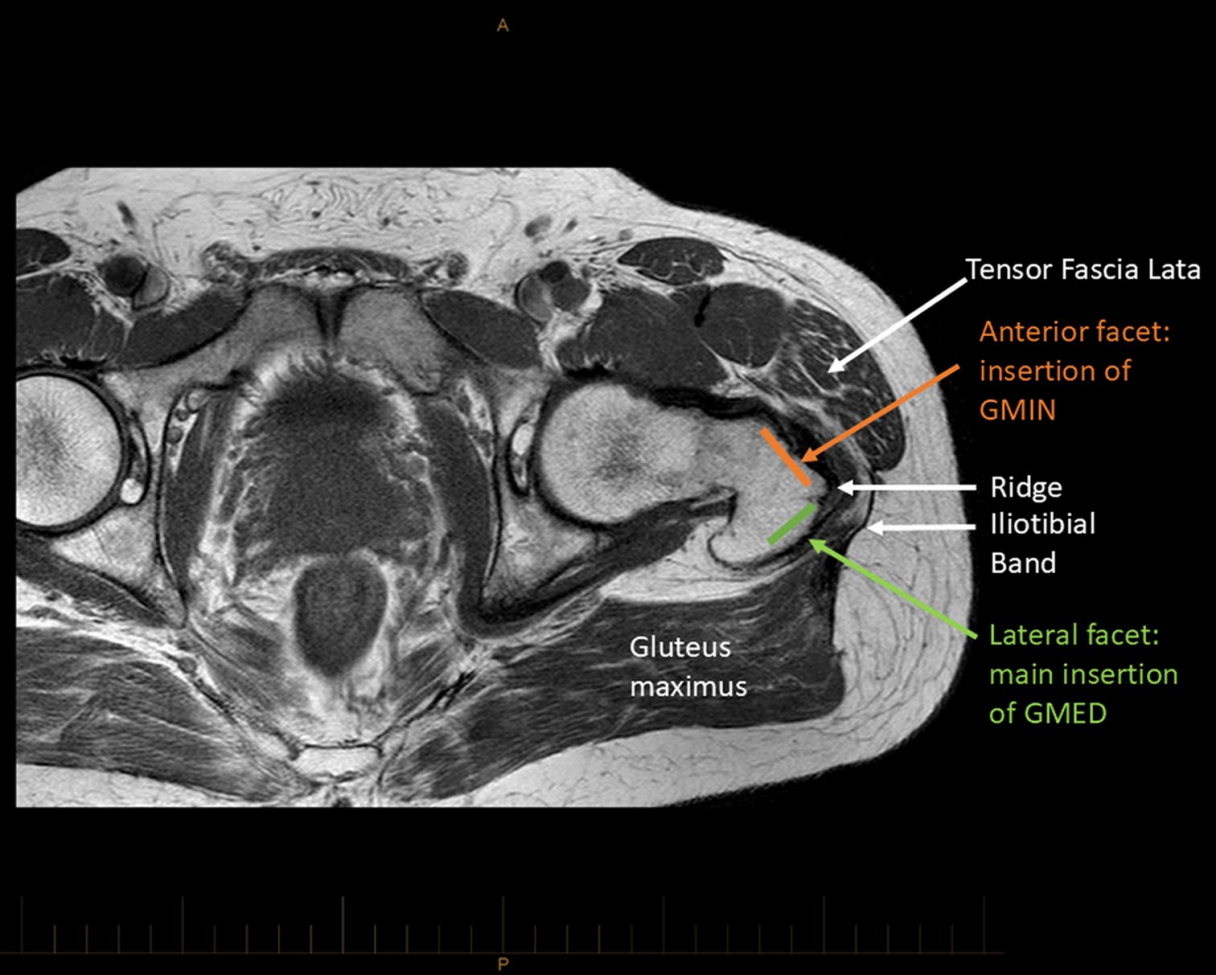
Axial cross-section of a T1 weighted MRI at the level of the greater trochanter, demonstrating the anterior and lateral facets of the greater trochanter, and the overlying iliotibial band and muscular tensioners - tensor fascia lata and gluteus maximus. GMED: Gluteus Medius; GMin: Gluteus Minimus

Awareness of the specific location of tendon pathology in those with GT may provide further insights into possible pathomechanical aspects that may be relevant in specific load management advice and exercise prescription. Our study found that pathology wasn’t equally distributed across tendon locations but appeared to be most prevalent within the anterior aspect of the GMed tendon and the posterior aspect of the GMin tendon, either side of the bony ridge separating the trochanteric facets into which they insert (Fig. 3). A recent ultrasound study reported that partial-thickness gluteal tendon tears were detected with the highest prevalence in the GMin tendon, followed by the more anterior GMed tendon, with tears in the most posterior aspect of the GMed tendon complex only present in 6.8% of participants [16], reflective of the pattern of tendon tear location present in the LEAP trial cohort.

Excessive compression at the insertion of the gluteal tendons is thought to be an important pathomechanical factor in those with GT [17]. Hip adduction is known to increase this compressive loading, with the taut iliotibial band compressing the tendons against the underlying bone [18]. When the tendons are tensioned over the greater trochanter in a position of hip adduction, the area of tendon draped over the apex of the trochanter is likely to be exposed to highest external compression. This may explain the high prevalence of pathology in the posterior aspect of the GMin and anterior aspect of the GMed tendons, where the anterior and lateral facets of the greater trochanter merge into a bony ridge [19, 20] (Fig. 3). Reducing exposure to excessive hip adduction or repetitive motion with the hip in an adducted position, may then reduce exposure to potentially adverse compressive and frictional tendon loads.

The LEAP education-and-exercise approach aimed to introduce graduated tensile loading of the abductor tendons in low-compression hip positions [6]. This included training participants to perform functional tasks such as walking, squatting, lunging, standing on one leg, and step-ups with reduced hip adduction [6]. In addition, participants were advised to avoid or minimise common compressive exercises for the region– hip adducted iliotibial band or gluteal stretches, and side-lying ‘clam’ or ‘clamshell’ type exercises [6].The finding of a focal region of gluteal tendon pathology at the trochanteric ridge, an area likely to be exposed to highest compressive and frictional loading, supports the approach taken in the LEAP protocol. Regardless of the severity of pathology, optimising mechanical loading across the trochanteric region is one element that may be helpful, within a broader biopsychosocial model of care.

The LEAP trial did not set out to specifically answer questions regarding the impact of interventions on outcomes in participants with varying levels of tendon pathology. There is no other RCT evidence currently available that has explored non-surgical management of gluteal tendon tears. This is an important direction for future research.

### Limitations

A strength of the study was the prospective and blinded data collection of MRIs from a large number of participants who had been clinically diagnosed with GT. It is important to recognise that we did not have partial or total avulsions of the GMed tendon in our cohort and suggest that clinicians consider this when interpreting our findings. A possible limitation was that a number of radiologists reported on the images and although they all used the same pre-determined reporting criteria, we did not evaluate inter-rater reliability and there is no reliability data evaluating the scoring method used in this study [4]. Future studies should consider using the Melbourne Hip MRI Score, to classify the severity of soft tissues change at the greater trochanter, as it has demonstrated excellent intra (ICC 0.81, 95% CI 0.67–0.89) and inter-observer reliability (ICC: 0.78, 95% CI 0.62–0.87) [21]. This scoring system was not available at the outset of the LEAP study. The pre-determined imaging assessment criteria reported hyperintensity but did not specifically report on bursal pathology. Future studies may also aim to specify the frequency of trochanteric, sub-gluteus medius and sub-gluteus minimus bursal pathology.

## Conclusion

A secondary analysis of a clinical trial that compared exercise-and-education with CSI and wait-and-see in 204 participants with imaging and clinically determined GT showed a substantial number of participants had advanced tendon pathology and that this was not associated with their reported levels of pain, function or disability. Specifically, over half of the participants included had pathological change in both the GMed and GMin tendons, around 40% had either partial or full-thickness tears in one or both gluteal tendons, and over one-third had calcific tendon deposits. As the outcome of the trial found a 77–78% success rate from an education-and-exercise approach in both the short and long-term, this suggests that such an approach may be an adequate first-line management strategy even for those with significant tendon changes. Prospective research is required to evaluate the impact of non-surgical interventions on outcomes in those with varying levels of gluteal tendon tears.

## Electronic supplementary material

Below is the link to the electronic supplementary material.

Supplementary Material 1

## Data Availability

All data supporting the findings of this study are available within the paper.
