# Supplementary material for: An examination of imaging findings in patients with clinically diagnosed gluteal tendinopathy: a secondary analysis of a randomised clinical trial
Source: Arch Orthop Trauma Surg. 2025 Jun 19;145(1):347. doi: 10.1007/s00402-025-05964-z (PMC12179227; doi:10.1007/s00402-025-05964-z)
Supplement: Supplementary file 1 — Supplementary Material 1 [file 402_2025_5964_MOESM1_ESM.docx]

Supplementary Appendix

This Supplementary Appendix has been provided to supply readers with additional information about this work.

Supplementary Table 1: Kellgren and Lawrence Categorisation of trial participants

| **KL scale category^** | **n (%)** |
| --- | --- |
| 0 = no OA | 116 (57) |
| 1 = doubtful | 49 (24) |
| 2 = minimal | 37 (18) |
| ^ KL = Kellgren and Lawrence System: no participants in categories 3 (moderate) and 4 (severe) | |

Supplementary Table 2: Correlation plots and statistics


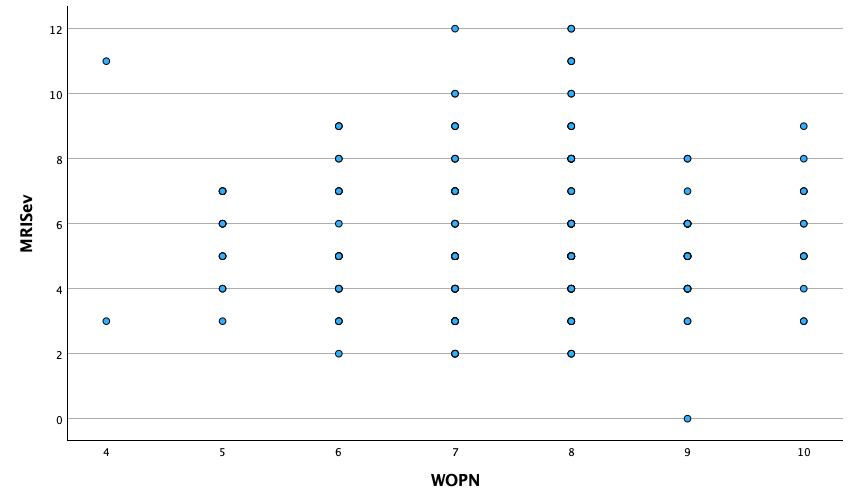


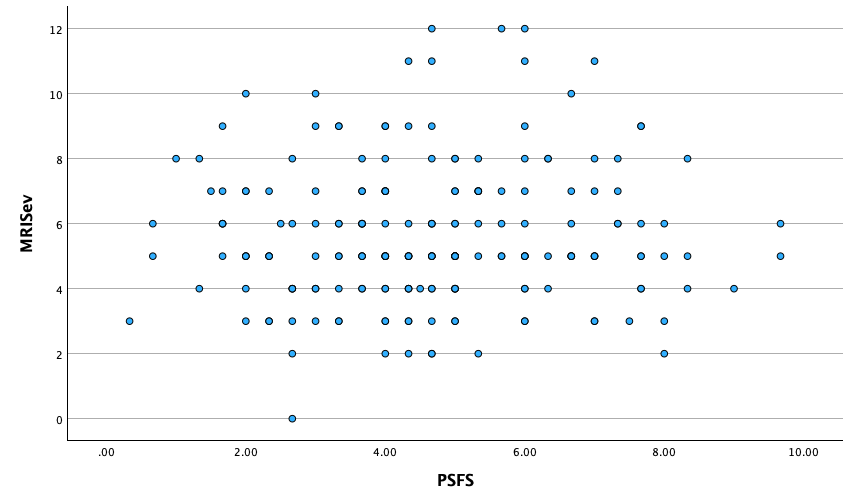


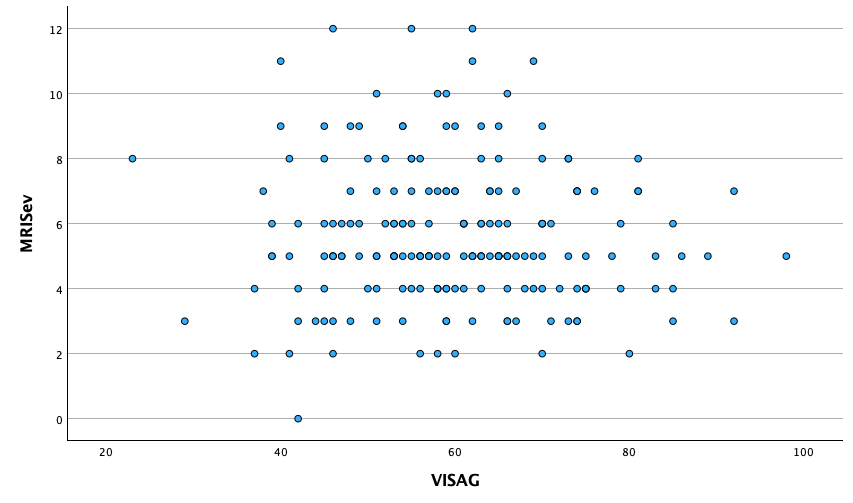


| **Correlations^b^** | | | | | |
| --- | --- | --- | --- | --- | --- |
|  | | MRISev | WOPN | VISAG | PSFS |
| MRISev | Pearson Correlation | 1 | .021 | -.038 | .042 |
|  | Sig. (2-tailed) |  | .771 | .596 | .566 |
| WOPN | Pearson Correlation | .021 | 1 | -.345^**^ | -.042 |
|  | Sig. (2-tailed) | .771 |  | <.001 | .563 |
| VISAG | Pearson Correlation | -.038 | -.345^**^ | 1 | .280^**^ |
|  | Sig. (2-tailed) | .596 | <.001 |  | <.001 |
| PSFS | Pearson Correlation | .042 | -.042 | .280^**^ | 1 |
|  | Sig. (2-tailed) | .566 | .563 | <.001 |  |
| **. Correlation is significant at the 0.01 level (2-tailed). | | | | | |
| b. Listwise N=193 | | | | | |
